# Extremely high HDL cholesterol paradoxically increases the risk of all-cause mortality in non-diabetic males from the Korean population: Korean genome and epidemiology study-health examinees (KoGES-HEXA) cohorts

**DOI:** 10.3389/fmed.2025.1534524

**Published:** 2025-05-15

**Authors:** Ha-Eun Ryu, Dong Hyuk Jung, Seok-Jae Heo, Byoungjin Park, Yong Jae Lee

**Affiliations:** ^1^Department of Family Medicine, Yongin Severance Hospital, Yongin, Gyeonggi-do, Republic of Korea; ^2^Department of Family Medicine, Yonsei University College of Medicine, Seoul, Republic of Korea; ^3^Biostatistics Collaboration Unit, Department of Biomedical Systems Informatics, Yonsei University College of Medicine, Seoul, Republic of Korea; ^4^Department of Family Medicine, Gangnam Severance Hospital, Seoul, Republic of Korea

**Keywords:** HDL cholesterol, all-cause mortality, cardiovascular risk factor, general population, HEXA cohort

## Abstract

**Background:**

High-density lipoprotein cholesterol (HDL-C) is associated with lower risk of mortality and cardiovascular disease. However, the relationship between extremely high HDL cholesterol level and all-cause mortality has not been thoroughly investigated. In this study, we examined the longitudinal effects of very high HDL cholesterol on all-cause mortality in a large cohort of Korean adults without type 2 diabetes mellitus.

**Methods:**

Data from 173,195 Korean participants over 40 years of age enrolled in the Korean Genome and Epidemiology Study-Health Examinees (KoGES-HEXA) cohort, linked with the death certificate database of the National Statistical Office, were assessed. Participants were grouped into four according to HDL-C levels. We used multivariate Cox proportional-hazard regression models to prospectively assess hazard ratios (HRs) for all-cause mortality with 95% confidence intervals (CIs) over an 11-year baseline period.

**Results:**

During a mean follow-up of 11.7 years, there were a total of 3,906 deaths from all causes, including 2,258 in men and 1,648 in women. The relationship between HDL-C and all-cause mortality showed a U-shaped pattern, especially in men. Compared to the reference group, the HR (95% CI) for mortality in males in the highest HDL cholesterol group was 1.31 (95% CI, 1.01–1.71) after adjusting for potential confounding variables. Moreover, low HDL cholesterol showed a statistically significant association with increased mortality in both men and women.

**Conclusion:**

Extremely high HDL-C levels could paradoxically increase the risk of all-cause mortality, particularly among males, in the general population without type 2 diabetes mellitus. Non-protective effects of very high HDL-C level should be noted when predicting incident metabolic syndrome, particularly in men, in clinical settings.

## Introduction

High-density lipoprotein cholesterol (HDL-C) has traditionally been regarded as a protective factor against cardiovascular disease (CVD). However, recent studies have challenged this idea, suggesting that extremely high HDL-C levels may paradoxically increase mortality risk (1–3). A U-shaped or J-shaped association between HDL-C and mortality has been observed, meaning that not only low but also very high HDL-C levels are associated with increased mortality risks. A prospective, multicenter cohort study using data from the UK general population and three Emory Healthcare sites in Atlanta, Georgia, found that individuals with coronary artery disease and HDL-C levels exceeding 80 mg/dL had a 96% higher risk of all-cause mortality and a 71% higher risk of cardiovascular mortality compared to those with HDL-C levels between 40 and 60 mg/dL, even after adjusting for covariates (1). Additionally, a population-based cohort study in the United States revealed a non-linear association between HDL-C levels and all-cause, cardiovascular, and cancer mortality in diabetic patients (2).

The contentious nature of these findings, particularly when stratified by sex, adds complexity to using HDL-C as a biomarker of the risk of CVD. The divergence in findings regarding the associations between HDL-C and mortality may stem from various factors, including sex-related factors. HDL-C levels tend to be higher in females than males (4), which is thought to be influenced by biological factors such as hormones. Indeed, HDL-C decreases in females after menopause (5). Differences among ethnic groups also exist, and lifestyle, behavioral factors, and genetic factors all contribute to variations in HDL-C levels (4). Moreover, these factors likely not only affect HDL-C levels but also impact other aspects of the body. Adjusting for these factors as comprehensively as possible to confirm associations between HDL-C and mortality is important.

The landscape of research on the association between HDL-C and mortality has been significantly influenced by studies focusing on specific risk groups, including individuals with type 2 diabetes mellitus (T2DM), atherosclerotic cardiovascular disease (ASCVD), and the older population (2, 6, 7). However, evaluating the association within specific risk groups can add an additional layer of complexity to understanding the role of HDL-C in mortality. In patients with T2DM, dyslipidemia, including of HDL-C, is a common finding. The prevalent lipid abnormality pattern in T2DM, referred to as diabetic dyslipidemia, encompasses elevated triglyceride levels, a diminished concentration of HDL-C, and a transition to small dense low-density lipoprotein (8). Hypertriglyceridemia leads to an increased level of triglyceride-enriched HDL, which undergoes accelerated clearance by the kidneys, ultimately resulting in decreased concentrations of HDL in the peripheral circulation (9). Furthermore, T2DM is characterized not only by reduced levels of HDL-C, but also impaired HDL function. This deficiency in functional HDL is closely linked to changes in the metabolism and structure of HDL within the vasculature (10, 11).

To date, research investigating the association between HDL and mortality in apparently healthy individuals without T2DM is still lacking. Therefore, our study aimed to explore the association between HDL and all-cause mortality in non-diabetic Koreans, focusing on sex differences.

## Materials and methods

### Study design and participants

**Figure 1:**
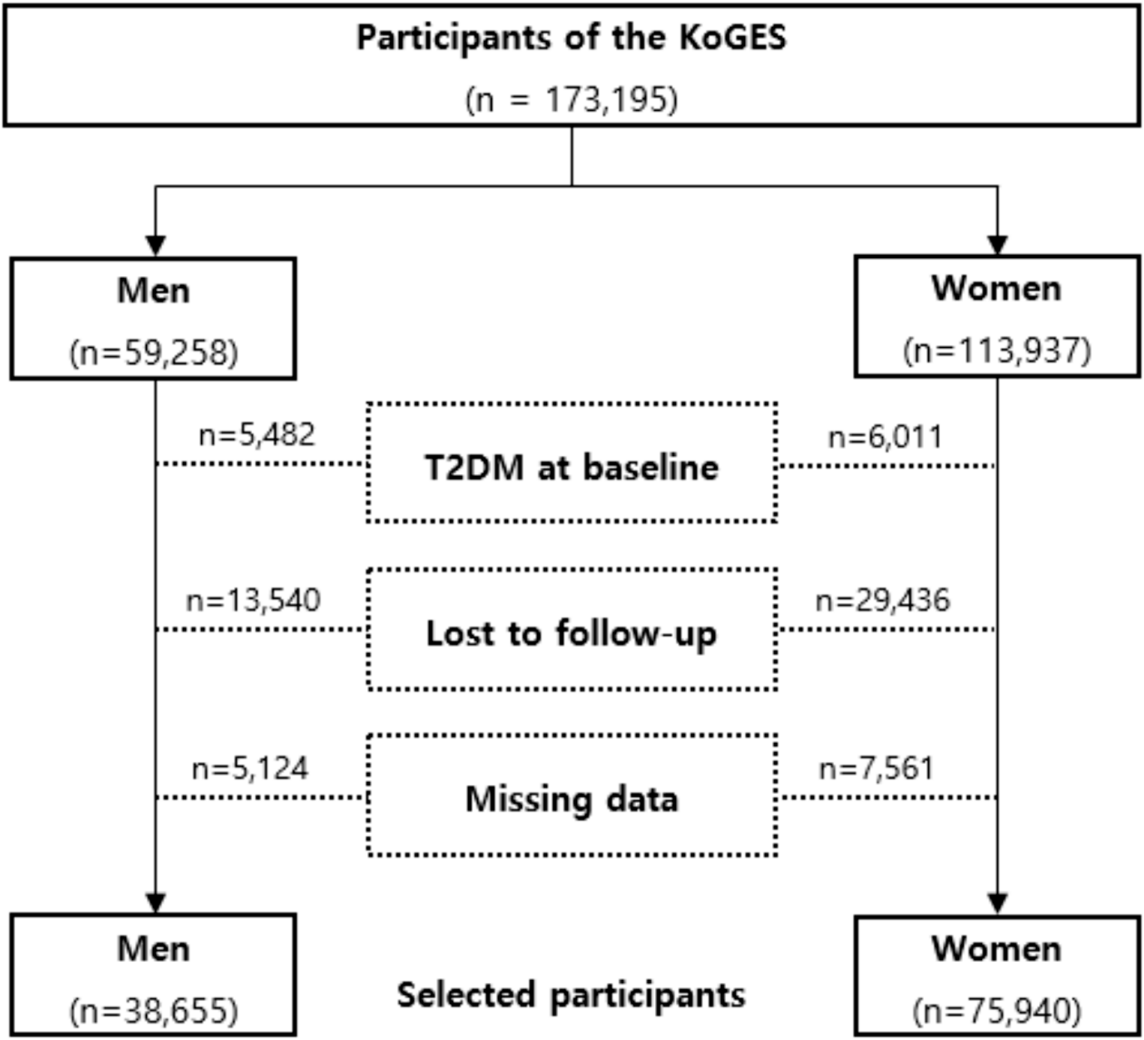
Flowchart of the study participants.

The Korean Genome and Epidemiologic Survey-Health Examinees (KoGES-HEXA) cohort is a key component of a large prospective study aimed at identifying genetic and environmental factors contributing to common complex diseases in Koreans, supported by government funding. The cohort consists of community residents and participants, both men and women aged 40 years or older at baseline, recruited from the National Health Examinee Registry. These individuals were enrolled during the baseline survey, conducted between 2004 and 2013, at 38 health examination centers and hospitals across eight regions in Korea. Participants in the follow-up were periodically invited to complete surveys via mail and telephone. For this analysis, anonymized data from 173,195 participants aged 40 and older were linked with the National Statistical Office’s death certificate database. The dataset for these participants includes anthropometric and clinical measurements, a lifestyle survey (covering diet, smoking, drinking, and physical activity), and a food frequency questionnaire. Participants were enrolled in 2004 and followed up between 2012 and 2018. Further details of the study have been published elsewhere [16]. This study investigated the general population without T2DM; a flow chart describing this study is presented in Figure 1. Among the 173,195 participants, we excluded 11,493 with pre-existing diabetes. Additionally, 42,976 participants lost to follow-up and 12,685 with missing covariate data were excluded. After these exclusions, 114,595 participants without diabetes were included, along with their disease history and mortality records. Our study adhered to the principles of the Declaration of Helsinki and was approved by the Ethics Committee of the Korean Health and Genomic Study at the Korea National Institute of Health. The HEXA study protocol was reviewed and approved by the Institutional Review Board of the Korea Centers for Disease Control and Prevention (now the Korea Disease Control and Prevention Agency), and all participants provided written informed consent. This research was approved by the Institutional Review Board of Yongin Severance Hospital, under IRB number 9–2023-0045.

### Data collection

Each participant completed a comprehensive questionnaire detailing their lifestyle and medical history. Smoking status was categorized as never-smoker, ex-smoker, or current smoker. Regular alcohol consumption was defined as drinking more than 140 grams per week, based on participants’ self-reported alcohol intake. Body weight and height were measured with an accuracy of 0.1 kg and 0.1 cm, respectively. Participants were instructed to wear light indoor clothing and to refrain from wearing shoes during the measurements. Body mass index (BMI) was calculated by dividing weight by height squared (kg/m^2^). Systolic blood pressure (SBP) and diastolic blood pressure (DBP) were assessed using a standard mercury sphygmomanometer (Baumanometer, W.A. Baum Co Inc., Copiague, NY, United States) while participants were seated and had rested for 10 min. Mean arterial pressure was derived from the measured SBP and DBP values. Hypertension was defined as having a SBP of ≥140 mmHg, a DBP of ≥90 mmHg, or current use of antihypertensive medication. Blood samples were collected from participants through an antecubital vein after a 12-h overnight fast. Concentrations of total cholesterol, HDL-C, low-density lipoprotein cholesterol (LDL-C), triglycerides, fasting plasma glucose, aspartate aminotransferase (AST) and alanine aminotransferase (ALT) were measured enzymatically using a chemistry analyzer (Hitachi 7,600, Tokyo, Japan, until August 2002; ADVIA 1650, Siemens, Tarrytown, NY, from September 2002).

### Study outcomes

Mortality status was determined by linking data to the unique personal identification key code system, as the HEXA cohort is connected to national data sources maintained by the Korea National Statistical Office, which contain mortality records. Participants were continuously followed from the baseline survey until the occurrence of a mortality event, the study end date, or the date of last contact. Mortality was monitored from January 2001 to December 2019, with causes of death classified according to International Classification of Diseases (ICD) codes in the National Mortality Index. All-cause mortality encompasses all deaths, whether from specified or unknown causes.

### Statistical analysis

We categorized participants into four groups based on their baseline HDL-C levels. Cut-off levels for HDL-C were 40, 60, and 80 mg/dL for men and 50, 75, and 100 mg/dL for women. All data presented in this study are expressed as means with standard deviations or frequencies with percentages. Baseline characteristics of the study population were compared across HDL-C quartiles using Pearson’s chi-squared test for categorical variables and analysis of variance (ANOVA) for continuous variables. Kaplan–Meier curves were employed to evaluate the cumulative incidence of all-cause mortality. Log-rank test was utilized to assess whether the distributions of the cumulative incidence function for mortality differed among groups. In multivariable analysis, using the lowest quartile of all-cause mortality as the reference group, hazard ratios (HRs) and 95% confidence intervals (CIs) for incident mortality were calculated using the Cox proportional hazards model, adjusted for potential confounding variables. All analyses were performed using R software (version 4.0.2; R Foundation for Statistical Computing, Vienna, Austria). All statistical tests were two-sided, and *p*-values less than 0.05 were considered statistically significant.

## Results

### Baseline characteristics

During a median follow-up period of 11.7 years, a total of 2,258 men and 1,648 women died. Table 1 presents the baseline characteristics of 38,655 men and 75,940 women categorized by HDL-C levels. Across both sexes, individuals in Group 4, characterized by the highest HDL-C levels, exhibited the lowest mean values for BMI, waist circumference, serum LDL-C, and triglyceride levels. Conversely, they had the highest mean values of fasting glucose, AST, and proportion of current drinkers. Conversely, individuals in Group 1, which had the lowest HDL-C levels, were older and had higher BMI, waist circumference, serum triglyceride, and ALT values. In addition, this group had the highest proportions of never drinkers, individuals with hypertension, chronic kidney disease, and individuals taking medications for hypertension. Additionally, the highest mean values of LDL-C were observed in Group 2 for both men and women. No significant differences were noted among groups regarding the proportion of individuals taking medications for dyslipidemia. Men in Group 4 had the highest mean SBP and DBP values, while women in Group 1 had the highest mean SBP and DBP values. Regarding smoking status, Group 1 had the highest proportion of current male smokers, whereas the majority of women were never smokers, with Group 1 having the highest proportion of never smokers among the study population.

**Table 1:** Baseline characteristics of the study population according to HDL quartiles in men and women.

Men	Group 1	Group 2	Group 3	Group 4	*p*-value
	HDL < 40 (*n* = 7,085)	HDL 40–60 (*n* = 25,027)	HDL 60–80 (*n* = 5,868)	HDL > 80 (*n* = 675)	
Age (years)	53.8 ± 8.6	53.3 ± 8.7	53.9 ± 8.7	54.8 ± 8.7	<0.001
Body mass index (kg/m^2^)	25.2 ± 2.7	24.4 ± 2.6	23.2 ± 2.7	22.3 ± 2.7	<0.001
Waist circumference (cm)	87.8 ± 7.0	85.6 ± 7.2	82.3 ± 7.6	79.8 ± 7.5	<0.001
Systolic blood pressure (mmHg)	124.8 ± 14.0	125.3 ± 14.3	125.4 ± 14.5	127.3 ± 15.0	<0.001
Diastolic blood pressure (mmHg)	77.9 ± 9.7	78.5 ± 9.7	78.5 ± 9.9	79.2 ± 9.9	<0.001
Fasting plasma glucose (mg/dL)	95.4 ± 16.1	95.2 ± 16.4	94.7 ± 16.1	95.8 ± 16.3	0.03
LDL cholesterol (mg/dL)	110.8 ± 31.0	118.8 ± 30.8	114.7 ± 30.4	108.1 ± 32.0	<0.001
Triglycerides (mg/dL)	180.4 ± 78.4	136.3 ± 69.5	100.3 ± 53.4	83.5 ± 41.2	<0.001
AST (IU/L)	25.5 ± 16.1	25.2 ± 13.7	26.4 ± 17.1	29.6 ± 20.7	<0.001
ALT (IU/L)	29.4 ± 25.4	26.4 ± 19.1	24.4 ± 20.6	23.7 ± 14.1	<0.001
Smoking status, *n* (%)					<0.001
Never smoker	1,897 (26.8)	7,058 (28.2)	1,634 (27.8)	179 (26.5)	
Former smoker	2,623 (37)	10,301 (41.2)	2,538 (43.3)	296 (43.9)	
Current smoker	2,565 (36.2)	7,668 (30.6)	1,696 (28.9)	200 (29.6)	
Alcohol intake, *n* (%)					<0.001
Never drinker	2,019 (28.5)	4,899 (19.6)	764 (13)	55 (8.1)	
Former drinker	642 (9.1)	1,620 (6.5)	272 (4.6)	25 (3.7)	
Current drinker	4,424 (62.4)	18,508 (74)	4,832 (82.3)	595 (88.1)	
Regular exercise, *n* (%)	3,709 (52.4)	14,262 (57)	3,546 (60.4)	407 (60.3)	<0.001
Hypertension, *n* (%)	1,668 (23.5)	5,078 (20.3)	1,034 (17.6)	113 (16.7)	<0.001
Chronic kidney disease, *n* (%)	174 (2.5)	295 (1.2)	39 (0.7)	3 (0.4)	<0.001
Diagnosis					
Cancer	172 (2.4)	530 (2.1)	157 (2.7)	14 (2.1)	0.048
Myocardial infarction	422 (6.0)	1,586 (6.3)	336 (5.7)	42 (6.2)	0.288
Thyroid disease	100 (1.4)	326 (1.3)	65 (1.1)	15 (2.2)	0.079
Medicine					
Hypertension, *n* (%)	1,432 (20.2)	4,258 (17)	832 (14.2)	96 (14.2)	<0.001
Dyslipidemia, *n* (%)	226 (3.2)	841 (3.4)	211 (3.6)	22 (3.3)	0.647

Women	Group 1	Group 2	Group 3	Group 4	*p*-value
	HDL < 50 (*n* = 5,481)	HDL 50–75 (*n* = 64,375)	HDL 75–100 (*n* = 5,806)	HDL > 100 (*n* = 278)	
Age (years)	54.3 ± 8.1	52.3 ± 7.9	50.9 ± 7.4	50.5 ± 6.7	<0.001
Body mass index (kg/m^2^)	24.6 ± 2.9	23.6 ± 2.9	22.3 ± 2.7	21.6 ± 2.5	<0.001
Waist circumference (cm)	81.2 ± 7.7	78.1 ± 8.0	74.2 ± 7.6	72.4 ± 7.3	<0.001
Systolic blood pressure (mmHg)	122.1 ± 15.2	120.3 ± 15.1	118.6 ± 14.5	120.3 ± 14.5	<0.001
Diastolic blood pressure (mmHg)	75.1 ± 9.8	74.4 ± 9.7	73.7 ± 9.5	74.4 ± 9.9	<0.001
Fasting plasma glucose (mg/dL)	92.4 ± 14.4	90.7 ± 13.7	89.3 ± 10.7	92.6 ± 17.4	<0.001
LDL cholesterol (mg/dL)	113.8 ± 31.9	122.8 ± 31.9	117.7 ± 30.1	110.9 ± 31.4	<0.001
Triglycerides (mg/dL)	168.4 ± 78.8	106.5 ± 55.2	73.2 ± 33.8	68.3 ± 30.3	<0.001
AST (IU/L)	23.5 ± 22.5	22.3 ± 11.8	22.8 ± 15.2	24.3 ± 8.9	<0.001
ALT (IU/L)	22.5 ± 36.5	19.5 ± 17.6	18.6 ± 19.5	18.7 ± 8.6	<0.001
Smoking status, *n* (%)					<0.001
Never smoker	5,291 (96.5)	62,142 (96.5)	5,532 (95.3)	257 (92.4)	
Former smoker	65 (1.2)	811 (1.3)	96 (1.7)	10 (3.6)	
Current smoker	125 (2.3)	1,422 (2.2)	178 (3.1)	11 (4)	
Alcohol intake, *n* (%)					<0.001
Never drinker	4,137 (75.5)	42,838 (66.5)	3,129 (53.9)	123 (44.2)	
Former drinker	105 (1.9)	1,254 (1.9)	85 (1.5)	5 (1.8)	
Current drinker	1,239 (22.6)	20,283 (31.5)	2,592 (44.6)	150 (54)	
Regular exercise, *n* (%)	2,438 (44.5)	32,306 (50.2)	3,226 (55.6)	170 (61.2)	<0.001
Hypertension, *n* (%)	1,278 (23.3)	10,018 (15.6)	626 (10.8)	33 (11.9)	<0.001
Chronic kidney disease, *n* (%)	94 (1.7)	393 (0.6)	36 (0.6)	1 (0.4)	<0.001
Diagnosis					
Cancer	238 (4.3)	2,357 (3.7)	231 (4.0)	16 (5.8)	0.014
Myocardial infarction	417 (7.6)	4,954 (7.7)	466 (8.0)	24 (8.6)	0.744
Thyroid disease	350 (6.4)	4,102 (6.4)	347 (6.0)	13 (4.7)	0.437
Medicine					
Hypertension, *n* (%)	1,137 (20.7)	8,571 (13.3)	532 (9.2)	29 (10.4)	<0.001
Dyslipidemia, *n* (%)	187 (3.4)	2,616 (4.1)	212 (3.7)	13 (4.7)	0.051

*P*-values were calculated using one-way ANOVA or Pearson’s chi-square test.

### Survival analysis of patients without T2DM with different HDL-C levels

**Figure 2:**
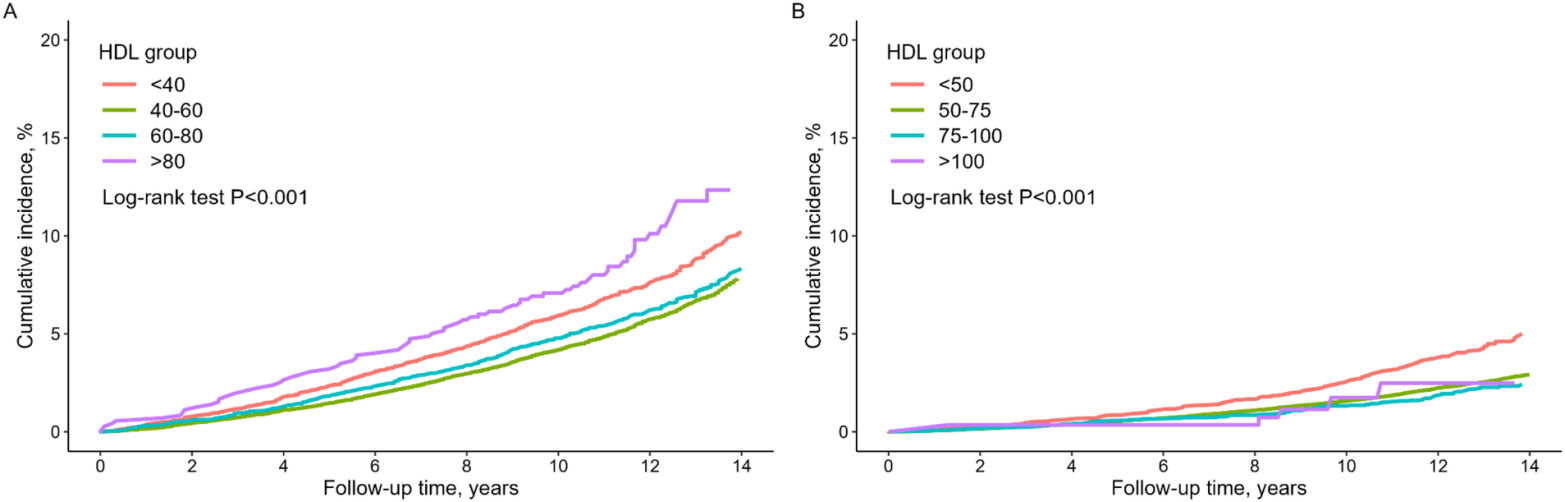
Kaplan–Meier curve for all-cause mortality by HDL groups. **(A)** Men, **(B)** women.

Figure 2 presents Kaplan–Meier curves demonstrating a significant difference in the cumulative survival rate among different HDL-C levels in both men and women (log-rank test, *p* < 0.001). Cumulative all-cause mortality rate of men in Group 4 was notably higher than that of men in the other groups, followed by Group 1. Conversely, women in Group 1 exhibited the highest cumulative all-cause mortality rate, significantly surpassing that of women in the other groups.

### Sex-specific relationships between HDL-C and mortality

**Figure 3:**
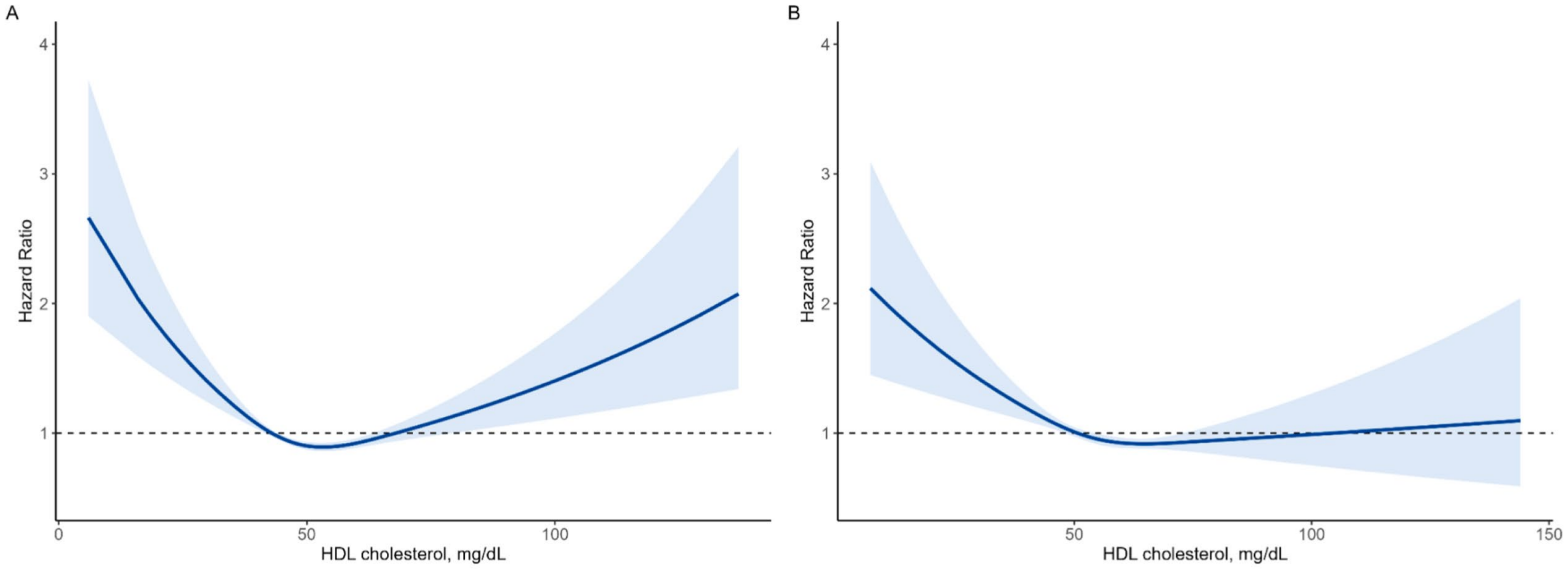
Non-linear association between HDL-C levels and all-cause mortality using a fully adjusted restricted cubic spline model. **(A)** Men, **(B)** women.

Spline analysis revealed U-curve associations between HDL-C levels and mortality in both men and women, as depicted in Figure 3. Spline analysis revealed U-curve associations between HDL-C levels and mortality in both men and women, as depicted in Figure 3. To further validate this non-linear association, we conducted a two-piecewise Cox proportional hazards model, identifying inflection points at 53.3 mg/dL in men and 64.7 mg/dL in women. Below these thresholds, HDL-C was inversely associated with mortality (HR = 0.83, 95% CI: 0.83–0.84 in men; HR = 0.89, 95% CI: 0.88–0.9 in women), whereas above these thresholds, higher HDL-C levels were associated with increased mortality risk (HR = 1.11, 95% CI: 1.11–1.12 in men; HR = 1.04, 95% CI: 1.03–1.05 in women). The log-likelihood ratio test confirmed that this model provided a significantly better fit than the standard Cox proportional hazards model (*p* < 0.001), further reinforcing the U-shaped relationship between HDL-C and mortality. These results are presented in Supplementary Table 1.

To further evaluate potential confounders, we assessed the associations of BMI, waist circumference, triglycerides, and alcohol intake with both HDL-C levels and all-cause mortality. All four factors were significantly associated with HDL-C levels in both men and women, while associations with all-cause mortality were observed only in men. These variables were included in our fully adjusted Cox models, and detailed results are presented in Supplementary Table 2.

Table 2 presents the results of sex-stratified Cox proportional hazard regression analysis for all-cause mortality based on HDL-C groups in men and women. Various models were constructed to assess the independent effect of HDL-C on all-cause mortality, adjusting for confounding variables. In Model 2, adjustments were made for age and BMI, while in Model 3, additional adjustments included waist circumference, SBP, DBP, fasting plasma glucose, LDL-C, triglycerides, smoking status, alcohol intake, AST, ALT, hypertension, chronic kidney disease, and medications for hypertension and dyslipidemia. Additionally, Model 4 included further adjustments for diagnosis of cancer, myocardial infarction, and thyroid disease.

**Table 2:** The sex-stratified Cox proportional hazard regression analysis results for all-cause mortality according to HDL groups in men and women.

Men		Group 1	Group 2	Group 3	Group 4
		HDL (<40)	HDL (40–60)	HDL (60–80)	HDL (>80)
All-cause mortality, n		486	1,349	363	60
Mean follow-up, years		11.8	11.9	11.7	11.4
Pearson-years of follow-up		83,661.1	297,532.3	68,880.4	7,662.8
Incidence rate/1,000 person-years		5.8	4.5	5.3	7.8
Model 1	HR (95% CI)	1.29 (1.16–1.43)	reference	1.16 (1.04–1.31)	1.76 (1.36–2.28)
	*p-*value	<0.001		0.011	<0.001
Model 2	HR (95% CI)	1.30 (1.17–1.45)	reference	1.03 (0.91–1.16)	1.42 (1.09–1.84)
	*p-*value	<0.001		0.634	0.009
Model 3	HR (95% CI)	1.22 (1.10–1.36)	reference	1.03 (0.91–1.16)	1.31 (1.01–1.71)
	*p-*value	<0.001		0.667	0.046
Model 4	HR (95% CI)	1.22 (1.09–1.36)	reference	1.03 (0.91–1.16)	1.32 (1.01–1.72)
	*p-*value	<0.001		0.672	0.040

Women		Group 1	Group 2	Group 3	Group 4
		HDL (<50)	HDL (50–75)	HDL (75–100)	HDL (>100)
All-cause mortality, *n*		189	1,361	92	6
Mean follow-up, years		12.0	12.0	11.7	10.9
Pearson-years of follow-up		65,959.5	774,645.0	68,041.0	3,028.0
Incidence rate/1,000 person-years		2.9	1.8	1.4	2.0
Model 1	HR (95% CI)	1.64 (1.41–1.91)	reference	0.78 (0.63–0.96)	1.20 (0.54–2.69)
	*p-*value	<0.001		0.021	0.649
Model 2	HR (95% CI)	1.32 (1.14–1.54)	reference	0.94 (0.76–1.16)	1.61 (0.72–3.59)
	*p-*value	<0.001		0.560	0.247
Model 3	HR (95% CI)	1.27 (1.08–1.49)	reference	0.91 (0.73–1.13)	1.44 (0.64–3.22)
	*p-*value	0.004		0.377	0.375
Model 4	HR (95% CI)	1.27 (1.08–1.49)	reference	0.90 (0.72–1.11)	1.37 (0.61–3.07)
	*p-*value	0.004		0.319	0.438

Model 1: crude.

Model 2: adjusted for age and body mass index.

Model 3: adjusted for age, body mass index, waist circumference, systolic blood pressure, diastolic blood pressure, fasting plasma glucose, low-density lipoprotein cholesterol, triglycerides, smoking status, alcohol intake, AST, ALT, hypertension, chronic kidney disease, and medicine for hypertension and dyslipidemia.

Model 4: adjusted for age, body mass index, waist circumference, systolic blood pressure, diastolic blood pressure, fasting plasma glucose, low density-lipoprotein cholesterol, triglyceride, smoking status, alcohol intake, AST, ALT, hypertension, chronic kidney disease, medicine for hypertension and dyslipidemia, diagnosis of cancer, myocardial infarction, and thyroid disease.

In men, the all-cause mortality risk in Group 4, characterized by the highest HDL-C level, significantly increased by 76, 42, 31, and 32% in models 1, 2, 3, and 4, respectively, compared to the reference group (*p* < 0.001, *p* = 0.009, *p* = 0.046, *p* = 0.040, respectively). Additionally, Group 1, with the lowest HDL-C level, exhibited notable increases in mortality risk by 29, 30, 22, and 22% in models 1, 2, 3, and 4, respectively, compared with the reference group (*p* < 0.001, all). Conversely, in women, only those in Group 1 displayed significant rises in mortality risk by 64, 32, 27, and 27% in models 1, 2, 3, and 4, respectively, relative to the reference group (*p* < 0.001, *p* < 0.001, *p* = 0.004, *p* = 0.004, respectively).

To further explore the relationship between HDL-C levels and specific causes of death, we conducted additional analyses using cardiovascular (CV)-related mortality and cancer-related mortality as outcomes. However, due to the limited number of events, no statistically significant associations were observed, particularly in the higher HDL-C groups. These results are presented in Supplementary Tables 3, 4.

### Subgroup analysis by age within each sex

We further conducted subgroup analyses stratified by age (≥60 years and <60 years) to explore potential heterogeneity in the association between HDL-C levels and all-cause mortality, as summarized in Table 3. Among males <60 years, both low (<40 mg/dL) and high (>80 mg/dL) HDL-C levels were significantly associated with increased mortality risk compared to the reference group (40–60 mg/dL), with HRs of 1.33 (95% CI: 1.12–1.58; *p* = 0.001) and 1.76 (95% CI: 1.19–2.60; *p* = 0.005), respectively. In males ≥60 years, only HDL-C < 40 mg/dL was significantly associated with higher mortality (HR 1.16; 95% CI: 1.01–1.34; *p* = 0.037). Among females, HDL-C < 40 mg/dL was significantly associated with higher mortality in the <60-year-old group (HR 1.42; 95% CI: 1.11–1.83; *p* = 0.006), while no significant associations were observed for HDL-C > 80 mg/dL in either age group.

**Table 3:** Subgroup analysis of hazard ratios for all-cause mortality according to HDL-C levels stratified by age and sex.

Men				
Group	Age ≥60 years		Age <60 years	
	HR (95% CI)	*p*-value	HR (95% CI)	*p*-value
HDL (<40)	1.16 (1.01–1.34)	0.037	1.33 (1.12–1.58)	0.001
HDL (40–60)	reference		reference	
HDL (60–80)	0.95 (0.81–1.10)	0.475	1.14 (0.94–1.38)	0.171
HDL (>80)	1.10 (0.76–1.57)	0.624	1.76 (1.19–2.60)	0.005

Women				
Group	Age ≥60 years		Age <60 years	
	HR (95% CI)	*p*-value	HR (95% CI)	*p*-value
HDL (<50)	1.21 (0.98–1.50)	0.075	1.42 (1.11–1.83)	0.006
HDL (50–75)	reference		reference	
HDL (75–100)	1.08 (0.79–1.46)	0.633	0.77 (0.57–1.05)	0.095
HDL (>100)	1.48 (0.37–5.97)	0.579	1.39 (0.52–3.72)	0.518

Adjusted for age, body mass index, waist circumference, systolic blood pressure, diastolic blood pressure, fasting plasma glucose, low density-lipoprotein cholesterol, triglyceride, smoking status, alcohol intake, AST, ALT, hypertension, chronic kidney disease, medicine for hypertension and dyslipidemia, diagnosis of cancer, myocardial infarction, and thyroid disease.

## Discussion

Our study revealed a U-shaped association between HDL-C levels and all-cause mortality in non-diabetic males. Both low HDL levels (<40 mg/dL) and very high HDL-C levels (>80 mg/dL) were linked to an increased risk of mortality compared with the reference group (40–60 mg/dL). Furthermore, the Kaplan–Meier curve for all-cause mortality by HDL-C group further emphasized that, in males, the very high HDL-C group experienced a higher incidence of mortality than the low HDL-C group (Figure 3). Conversely, in females, when compared to the reference group (50–75 mg/dL), there was a significant association with increased mortality in the lower HDL-C range (<50 mg/dL), while no significant association was observed in the higher HDL-C range (>75 mg/dL).

An increase in HDL-C levels has been associated with a decrease in ASCVD risk, leading to its classification as “good” cholesterol (12, 13). In more detail, in the ASCVD risk prediction model proposed by the Framingham Heart Study, an HDL-C level ≥ 60 mg/dL results in a reduction of 1 when performing the ASCVD risk factor calculation. This implies that maintaining HDL-C levels above this threshold has cardio-protective benefits (14). Based on this, there has been considerable interest in raising HDL levels with the anticipation of a subsequent reduction in ASCVD risk. However, the results have been somewhat disappointing. In randomized controlled trials evaluating the effects of inhibiting the cholesteryl ester transfer protein (CETP) to elevate HDL-C levels (15, 16), no reduction in CVD risk was observed and in some cases, the trial was prematurely terminated due to an increase in mortality rate (15). Additionally, numerous epidemiological studies have consistently revealed a U-shaped association between HDL and mortality (17–22). This has raised questions about whether HDL is indeed the “good cholesterol” and has underscored the necessity for reevaluation of its purported cardiovascular protective effects.

Various factors can contribute to elevated HDL-C levels, including genetic variants such as CETP deficiency and mutations in ABCA1 or APOA1, as well as lifestyle factors like moderate-to-high alcohol consumption, regular physical activity, and low triglyceride levels (23–25). In our study, individuals in the highest HDL-C quartile had lower BMI, smaller waist circumference, and higher rates of alcohol intake and exercise (Table 1). Additional linear regression analyses confirmed significant associations between HDL-C levels and BMI, waist circumference, triglycerides, and alcohol intake in both sexes (Supplementary Table 2). However, elevations in HDL-C from these factors do not necessarily indicate improved HDL function and may instead be associated with dysfunctional or pro-inflammatory HDL particles (26), which could help explain the increased mortality risk observed at extremely high HDL-C levels.

The mechanisms underlying the paradoxical increase in mortality observed at extremely high levels of HDL-C remain incompletely understood. However, several plausible explanations can be considered, including the loss of the protective functions of HDL, genetic influences, and disruptions in cholesterol transport dynamics. The conventional understanding of the cardioprotective role of HDL-C primarily centers on reverse cholesterol transport, wherein HDL facilitates the efflux of cholesterol from peripheral tissues and atherosclerotic plaques to the liver for excretion, thereby reducing the risk of ASCVD (27). However, recent evidence suggests that excessively high HDL-C levels may be associated with dysfunctional HDL particles that lose their protective properties and may even exert harmful effects (26). HDL particles are structurally diverse, differing in size, density, and apolipoprotein content, which influences their functional capacity (4). In an oxidative or inflammatory environment, HDL can become pro-inflammatory and pro-atherogenic, impairing endothelial function and promoting vascular dysfunction (28). Additionally, oxidative modifications of apolipoprotein A-I, the major protein component of HDL, have been shown to reduce cholesterol efflux efficiency, limiting the anti-atherogenic properties of HDL (15). Thus, in individuals with extremely high HDL-C levels, it is possible that HDL particles undergo structural and functional alterations that negate their vascular protective effects, thereby increasing mortality risk.

In addition to functional impairments, genetic factors regulating HDL-C metabolism may contribute to the increased mortality risk associated with very high HDL-C. Several genetic variants have been identified that lead to markedly elevated HDL-C concentrations and may exert systemic effects beyond simply increasing HDL-C levels (25). Indeed, some of these variants have been associated with an increased risk of cardiovascular disease despite high HDL-C levels, as demonstrated in previous studies (29–32). Thus, the associations observed in this study might be partially explained by underlying genetic determinants that predispose individuals with extremely high HDL-C to increased mortality risks. Furthermore, the reverse remnant cholesterol transport hypothesis offers additional insight into the non-linear relationship between HDL-C and mortality. According to this hypothesis, free cholesterol from triglyceride-rich lipoproteins is transferred to HDL for removal. However, previous research has shown that this transfer process becomes less efficient at both very low and very high HDL-C levels, leading to impaired cholesterol clearance (33). As a result, cholesterol-rich remnant particles may accumulate, contributing to a higher risk of cardiovascular and all-cause mortality. This may help explain the U-shaped relationship observed between HDL-C levels and mortality.

While our primary focus was on all-cause mortality, we also examined CV-related and cancer-related mortality to assess whether the association with HDL-C levels varied by cause of death. However, no significant associations were observed, particularly among individuals with higher HDL-C levels. These findings suggest that the impact of HDL-C on mortality may be mediated by multiple underlying mechanisms rather than a single dominant cause. Given prior research suggesting that very high HDL-C may be more strongly associated with non-cardiovascular mortality (17, 34), future studies with larger datasets and more detailed cause-of-death classifications are needed to better elucidate these associations.

In our study, we did not observe a significant association between high HDL and mortality in women, unlike in men. Women generally have higher levels of HDL-C than men, which could be a factor contributing to the lack of a distinct association with mortality (4). Guidelines for CVD prevention also divide the criteria for low HDL-C associated with elevated CVD risk by sex, setting it at <40 mg/dL in men and <50 mg/dL in women (35, 36). We applied this division when categorizing HDL-C groups for men and women, respectively. There has been inconsistency in previous studies regarding the association between very high HDL cholesterol and mortality outcomes, particularly when stratified by sex. Several prior studies, similar to ours, found a significant positive association between high HDL and mortality in males. However, these studies also failed to find a significant association between high HDL and mortality in females (6, 17, 20, 37). However, other studies have reported increased mortality risk associated with very high HDL-C in both sexes. For example, a large-scale study that included two prospective cohort studies demonstrated a U-shaped association between both extremely high and low concentrations of HDL-C and elevated all-cause mortality risk in both males and females (38). This study, conducted in a Danish population, suggests that ethnic differences might contribute to the variance in results, considering that Asian Americans typically have lower levels of HDL-C than white people (39). Another significant study in Canada, involving a big data approach with 631,762 participants without previous cardiovascular conditions, reported that individuals with elevated HDL levels (>70 mg/dL in men, >90 mg/dL in women) had a higher risk of non-cardiovascular mortality (34). It is crucial to note the distinction from our study, which focused on all-cause mortality. Finally, research conducted on Koreans with ASCVD demonstrated that both males and females in the extremely high HDL-C (>90 mg/dL) category had 35.9 and 9.5% higher 10-year mortality risks than those in the high HDL-C group (40–90 mg/dL for males, 50–90 mg/dL for females), respectively (7). However, it is essential to recognize that this study specifically targeted individuals with ASCVD, unlike our study population.

In subgroup analyses stratified by age, the association between HDL-C levels and all-cause mortality differed across age and sex. Among males under 60 years, both low (<40 mg/dL) and high (>80 mg/dL) HDL-C levels were significantly associated with increased mortality, consistent with the overall U-shaped pattern observed in the main analysis. In contrast, among males aged 60 years or older, only low HDL-C was associated with higher mortality, while extremely high HDL-C showed no significant association. In females, low HDL-C was linked to increased mortality only in those under 60, and no significant association was observed for high HDL-C in either age group. These findings suggest that younger individuals, particularly males, may be more vulnerable to the adverse effects of extremely high HDL-C, while this relationship weakens with age. One possible explanation involves age-related changes in lipoprotein metabolism and HDL functionality. Aging alters HDL composition—reducing cholesterol and apoE content, increasing sphingomyelin, and enriching pro-inflammatory proteins such as serum amyloid A—which impairs its antioxidant capacity and paraoxonase 1 activity (40). These changes may convert HDL from an anti-atherogenic to a pro-atherogenic particle. Additionally, age-related endothelial dysfunction and chronic inflammation may amplify the harmful effects of dysfunctional HDL in younger adults, while in older individuals, the cumulative burden of comorbidities may overshadow HDL-related risk. These results underscore the importance of considering age and sex when interpreting HDL-C levels and highlight the need for individualized lipid management strategies.

Our study has certain limitations. First, although we conducted additional analyses examining CV-related and cancer-related mortality, these did not yield statistically significant findings, likely due to the limited number of events. Additionally, data collection in our study focused on Korean adults aged 40–69 years, which may limit the applicability of our findings to other countries, age ranges, and ethnic groups. Second, our study was limited to Korean adults aged 40–69 years, which may restrict the generalizability of our findings to other populations. Ethnic and regional variations in genetic predisposition, dietary habits, lifestyle factors, and healthcare systems may influence the association between HDL-C and mortality (39). Therefore, validation in diverse racial and geographic populations is essential to determine whether these associations are consistent across different ethnic backgrounds. Future large-scale, multi-ethnic cohort studies are needed to further clarify these relationships and refine risk stratification for individuals with extremely high HDL-C levels. Finally, there may have been unaccounted-for residual confounding factors. The strength of our study lies in its exploration of the association between HDL-C and mortality in a relatively healthy population, excluding the confounding influence of T2DM. Particularly noteworthy is the insight from our study that in males, when HDL-C is above 80 mg/dL, the cumulative incidence of mortality is higher than the commonly recognized risk factor of low HDL-C, emphasizing the urgency of setting upper-level thresholds. Additional research is essential to determine at what concentration HDL-C loses its protective functions. Ongoing research is imperative to unravel the complexities of HDL-C’s roles in the human body (41–43).

## Conclusion

We demonstrated a U-shaped correlation between HDL-C levels and all-cause mortality in non-diabetic Korean males, but not females. Furthermore, we observed that in males, the very high HDL-C group (>80 mg/dL) had a higher cumulative incidence of mortality than the low HDL-C group (<40 mg/dL). Further research is urgently needed, particularly in males, to establish upper-level thresholds for HDL-C.

## Data Availability

Publicly available datasets were analyzed in this study. This data can be found: the dataset used in this study was obtained after the review and evaluation of the research plan by the Korea Centers for Disease Control and Prevention.
