# Supplementary material for: Extremely high HDL cholesterol paradoxically increases the risk of all-cause mortality in non-diabetic males from the Korean population: Korean genome and epidemiology study-health examinees (KoGES-HEXA) cohorts
Source: Front Med (Lausanne). 2025 May 15;12:1534524. doi: 10.3389/fmed.2025.1534524 (PMC12119502; doi:10.3389/fmed.2025.1534524)
Supplement: Supplementary file 1 [file Table_1.docx]

**Supplementary Table 1.** Two-piecewise Cox proportional hazards model examining the non-linear association between HDL-C levels and all-cause mortality, with identified inflection points for men and women.

| **Male** |  |  |
| --- | --- | --- |
| **Fitting model by standard cox proportional hazard model** | | |
| HR (95% CI) per 10 mg/dL | 0.98 (0.97-0.98) | |
| **Fitting model by two-piecewise cox proportional hazard model** | | |
| Infection point of HDL (mg/dL) | 53.3 | |
| HR (95% CI) per 10 mg/dL |  | |
| <53.3 | 0.83 (0.83-0.84) | |
| ≥53.3 | 1.11 (1.1-1.12) | |
| P for log likelihood ratio test | <0.001 | |

| **Female** |  |  |
| --- | --- | --- |
| **Fitting model by standard cox proportional hazard model** | | |
| HR (95% CI) per 10 mg/dL | 0.94 (0.93-0.94) | |
| **Fitting model by two-piecewise cox proportional hazard model** | | |
| Infection point of HDL (mg/dL) | 64.7 | |
| HR (95% CI) per 10 mg/dL |  | |
| <64.7 | 0.89 (0.88-0.9) | |
| ≥64.7 | 1.04 (1.03-1.05) | |
| P for log likelihood ratio test | <0.001 | |

**Supplementary Table 2.** Associations of body mass index, waist circumference, triglyceride levels, and alcohol intake with HDL-C levels (linear regression) and with all-cause mortality (Cox proportional hazards models), stratified by sex.

| Variable | Linear regression for HDL | | | |
| --- | --- | --- | --- | --- |
|  | Male | | Female | |
|  | Beta (95% CI) | p-value | Beta (95% CI) | p-value |
| Body mass index (kg/m2) | -0.44 (-0.50,-0.37) | <0.001 | -0.27 (-0.31,-0.22) | <0.001 |
| Waist circumference (cm) | -0.18 (-0.21,-0.16) | <0.001 | -0.18 (-0.20,-0.16) | <0.001 |
| Triglycerides (mg/dL) | -0.05 (-0.06,-0.05) | <0.001 | -0.08 (-0.09,-0.08) | <0.001 |
| Alcohol intake, n (%) |  |  |  |  |
| Never drinker | reference |  | reference |  |
| Former drinker | 0.27 (-0.20,0.75) | 0.257 | 0.16 (-0.44,0.76) | 0.600 |
| Current drinker | 4.52 (4.25,4.79) | <0.001 | 2.93 (2.75,3.11) | <0.001 |

The linear regression model included age, body mass index, waist circumference, systolic blood pressure, diastolic blood pressure, fasting plasma glucose, low density-lipoprotein cholesterol, triglyceride, smoking status, alcohol intake, AST, ALT, hypertension, chronic kidney disease, medicine for hypertension and dyslipidemia, diagnosis of cancer, myocardial infarction, and thyroid disease as independent variables.

| Variable | Cox proportional hazard model for all-cause mortality | | | |
| --- | --- | --- | --- | --- |
|  | Male | | Female | |
|  | HR (95% CI) | p-value | HR (95% CI) | p-value |
| Body mass index (kg/m2) | 0.94 (0.91-0.96) | <0.001 | 0.99 (0.96-1.02) | 0.463 |
| Waist circumference (cm) | 1.01 (1.00-1.02) | 0.004 | 1.01(1.00-1.02) | 0.182 |
| Triglycerides (mg/dL) | 0.99 (0.98-1.00) | <0.001 | 1.00 (0.99-1.00) | 0.345 |
| Alcohol intake, n (%) |  |  |  |  |
| Never drinker | reference |  | reference |  |
| Former drinker | 1.33 (1.15-1.55) | <0.001 | 1.24 (0.92-1.68) | 0.160 |
| Current drinker | 0.92 (0.82-1.02) | 0.101 | 0.91 (0.80-1.03) | 0.122 |

The Cox proportional hazard model included HDL-C, age, body mass index, waist circumference, systolic blood pressure, diastolic blood pressure, fasting plasma glucose, low density-lipoprotein cholesterol, triglyceride, smoking status, alcohol intake, AST, ALT, hypertension, chronic kidney disease, medicine for hypertension and dyslipidemia, diagnosis of cancer, myocardial infarction, and thyroid disease as independent variables.

**Supplementary Table 3.** Cox proportional hazard regression analysis results for cardiovascular-related mortality according to HDL-C groups in men and women.

| Men | | Group 1 | Group 2 | Group 3 | Group 4 |
| --- | --- | --- | --- | --- | --- |
|  |  | HDL(<40) | HDL(40-60) | HDL(60-80) | HDL(>80) |
| **CV**-related mortality, n | | 89 | 210 | 50 | 8 |
| Mean follow-up, years | | 11.8 | 11.9 | 11.7 | 11.4 |
| Pearson-years of follow-up | | 83661.1 | 297532.3 | 68880.4 | 7662.8 |
| Incidence rate/1000 person-years | | 1.06 | 0.71 | 0.73 | 1.04 |
| Model 1 | HR (95% CI) | 1.52 (1.18-1.95) | reference | 1.03 (0.76-1.40) | 1.51 (0.74-3.05) |
|  | *p* value | <0.001 |  | 0.849 | 0.255 |
| Model 2 | HR (95% CI) | 1.52 (1.18-1.94) | reference | 0.92 (0.68-1.26) | 1.25 (0.61-2.54) |
|  | *p* value | 0.001 |  | 0.615 | 0.544 |
| Model 3 | HR (95% CI) | 1.37 (1.06-1.79) | reference | 0.95 (0.69-1.30) | 1.23 (0.60-2.53) |
|  | *p* value | 0.018 |  | 0.733 | 0.574 |
| Model 4 | HR (95% CI) | 1.37 (1.05-1.78) | reference | 0.95 (0.69-1.30) | 1.24 (0.60-2.54) |
|  | *p* value | 0.019 |  | 0.744 | 0.565 |
|  | | | | | |

| Women | | Group 1 | Group 2 | Group 3 | Group 4 |
| --- | --- | --- | --- | --- | --- |
|  |  | HDL(<50) | HDL(50-75) | HDL(75-100) | HDL(>100) |
| **CV**-related mortality, n | | 34 | 222 | 14 | 0 |
| Mean follow-up, years | | 12.03421 | 12.03332 | 11.71909 | 10.89206 |
| Pearson-years of follow-up | | 65959.493 | 774644.95 | 68041.013 | 3027.992 |
| Incidence rate/1000 person-years | | 0.52 | 0.29 | 0.21 | 0 |
| Model 1 | HR (95% CI) | 1.81 (1.26-2.60) | reference | 0.73 (0.42-1.25) | - |
|  | *p* value | 0.001 |  | 0.251 |  |
| Model 2 | HR (95% CI) | 1.35 (0.94-1.94) | reference | 0.98 (0.57-1.69) | - |
|  | *p* value | 0.104 |  | 0.943 |  |
| Model 3 | HR (95% CI) | 1.25 (0.85-1.83) | reference | 0.96 (0.55-1.67) | - |
|  | *p* value | 0.265 |  | 0.889 |  |
| Model 4 | HR (95% CI) | 1.25 (0.85-1.83) | reference | 0.97 (0.56-1.68) | - |
|  | *p* value | 0.262 |  | 0.909 |  |
| Model 1: crude | | | | | |
| Model 2: adjusted for age and body mass index, | | | | | |
| Model 3: adjusted for age, body mass index, waist circumference, systolic blood pressure, diastolic blood pressure, fasting plasma glucose, low-density lipoprotein cholesterol, triglycerides, smoking status, alcohol intake, AST, ALT, hypertension, chronic kidney disease, and medicine for hypertension and dyslipidemia. | | | | | |
| Model 4: adjusted for age, body mass index, waist circumference, systolic blood pressure, diastolic blood pressure, fasting plasma glucose, low density-lipoprotein cholesterol, triglyceride, smoking status, alcohol intake, AST, ALT, hypertension, chronic kidney disease, medicine for hypertension and dyslipidemia, diagnosis of cancer, myocardial infarction, and thyroid disease. | | | | | |

**Supplementary Table 4.** Cox proportional hazard regression analysis results for cancer-related mortality according to HDL-C groups in men and women.

| Men | | Group 1 | Group 2 | Group 3 | Group 4 |
| --- | --- | --- | --- | --- | --- |
|  |  | HDL(<40) | HDL(40-60) | HDL(60-80) | HDL(>80) |
| **Cancer**-related mortality, n | | 236 | 614 | 155 | 24 |
| Mean follow-up, years | | 11.8082 | 11.88845 | 11.73831 | 11.35227 |
| Pearson-years of follow-up | | 83661.101 | 297532.345 | 68880.411 | 7662.782 |
| Incidence rate/1000 person-years | | 2.82 | 2.06 | 2.25 | 3.13 |
| Model 1 | HR (95% CI) | 1.37 (1.18-1.60) | reference | 1.09 (0.91-1.30) | 1.54 (1.02-2.31) |
|  | *p* value | <0.001 |  | 0.332 | 0.038 |
| Model 2 | HR (95% CI) | 1.36 (1.17-1.59) | reference | 1.00 (0.83-1.19) | 1.30 (0.86-1.97) |
|  | *p* value | <0.001 |  | 0.963 | 0.206 |
| Model 3 | HR (95% CI) | 1.30 (1.11-1.52) | reference | 0.98 (0.81-1.17) | 1.17 (0.77-1.77) |
|  | *p* value | 0.001 |  | 0.801 | 0.463 |
| Model 4 | HR (95% CI) | 1.29 (1.10-1.52) | reference | 0.97 (0.81-1.17) | 1.18 (0.78-1.79) |
|  | *p* value | 0.001 |  | 0.784 | 0.432 |
|  | | | | | |

| Women | | Group 1 | Group 2 | Group 3 | Group 4 |
| --- | --- | --- | --- | --- | --- |
|  |  | HDL(<50) | HDL(50-75) | HDL(75-100) | HDL(>100) |
| **Cancer**-related mortality, n | | 103 | 727 | 36 | 3 |
| Mean follow-up, years | | 12.03421 | 12.03332 | 11.71909 | 10.89206 |
| Pearson-years of follow-up | | 65959.493 | 774644.95 | 68041.013 | 3027.992 |
| Incidence rate/1000 person-years | | 1.56 | 0.94 | 0.53 | 0.99 |
| Model 1 | HR (95% CI) | 1.66 (1.35-2.05) | reference | 0.57 (0.41-0.80) | 1.12 (0.36-3.47) |
|  | *p* value | <0.001 |  | 0.001 | 0.85 |
| Model 2 | HR (95% CI) | 1.40 (1.13-1.72) | reference | 0.67 (0.48-0.93) | 1.40 (0.45-4.34) |
|  | *p* value | 0.002 |  | 0.018 | 0.565 |
| Model 3 | HR (95% CI) | 1.43 (1.15-1.78) | reference | 0.63 (0.45-0.89) | 1.24 (0.40-3.87) |
|  | *p* value | 0.001 |  | 0.008 | 0.711 |
| Model 4 | HR (95% CI) | 1.44 (1.15-1.79) | reference | 0.62 (0.44-0.87) | 1.16 (0.37-3.62) |
|  | *p* value | 0.001 |  | 0.006 | 0.797 |
| Model 1: crude | | | | | |
| Model 2: adjusted for age and body mass index, | | | | | |
| Model 3: adjusted for age, body mass index, waist circumference, systolic blood pressure, diastolic blood pressure, fasting plasma glucose, low-density lipoprotein cholesterol, triglycerides, smoking status, alcohol intake, AST, ALT, hypertension, chronic kidney disease, and medicine for hypertension and dyslipidemia. | | | | | |
| Model 4: adjusted for age, body mass index, waist circumference, systolic blood pressure, diastolic blood pressure, fasting plasma glucose, low density-lipoprotein cholesterol, triglyceride, smoking status, alcohol intake, AST, ALT, hypertension, chronic kidney disease, medicine for hypertension and dyslipidemia, diagnosis of cancer, myocardial infarction, and thyroid disease. | | | | | |
